# Differential Annual Movement Patterns in a Migratory Species: Effects of Experience and Sexual Maturation

**DOI:** 10.1371/journal.pone.0022433

**Published:** 2011-07-20

**Authors:** Paulo E. Jorge, David Sowter, Paulo A. M. Marques

**Affiliations:** 1 Unidade de Investigação em Eco-Etologia, ISPA Instituto Universitário, Lisboa, Portugal; 2 Department of Biological Sciences, Virginia Tech, Blacksburg, Virginia, United States of America; 3 The NW Gull Project, Penwortham, Preston, United Kingdom; 4 Museu Nacional História Natural, Universidade Lisboa, Lisboa, Portugal; University of Plymouth, United Kingdom

## Abstract

Some animals migrate long distances to exploit important seasonal food resources in the northern regions of the northern hemisphere, whilst avoiding winter starvation. Changes in the individual's age and navigational skills are likely to affect migration, which in turn influences the geographic distribution of individuals. Processes such as sexual maturation and navigational abilities are affected by age, and age is thus a key factor in understanding migration patterns and differences in distribution ranges. In the present study, we investigated the effects of age on the geographic distribution of a population of Lesser Black-backed Gulls *Larus fuscus* throughout its annual cycle, by analyzing a dataset of 19,096 records from 10,000 color-ringed gulls. In contrast to previous assumptions, the results showed that gulls were geographically segregated by age throughout the entire annual cycle, rather than showing a geographic age-related cline only in the wintering areas. This asymmetric distribution results from a reduction in the annual range of sexually mature gulls, and the differential distribution of mature and immature individuals (mature birds remained in more northern areas, compared to immature birds, throughout the annual cycle). Furthermore, although immature gulls travelled longer distances than adults, they initiated their fall migration with short movements, in contrast to adults that migrated using longer movements. The effects identified in this study explain the non-homogenous distribution of populations throughout the annual cycle, with wide implications for the development of effective human health policies and/or wildlife management strategies.

## Introduction

The ability to move through a medium (such as air, water or mud) is a feature common to many living organisms. This movement can range from a few millimeters (e.g., dispersal of bacterial colonies [1]) to thousands of kilometers (e.g., circumpolar migration of arctic terns [2]), and plays an important role in the survival of organisms, not only at the population level, but also at the ecosystem [3] and evolutionary [4] levels.

There have been considerable advances in our understanding of the dynamics of migration over the last 50 years [5], [6], pointing to the importance of the individual's sex or age or both on its annual movement. Evidence for differential migration patterns within a population has been proposed for several species [7], but these patterns have generally been associated with particular seasons of the year (e.g., fall migration) or specific goals (e.g., feeding areas). However, a need to study annual movements has recently emerged, because it is now known that temporally distinct life-history events [8] e.g., breeding and wintering-range variation [9], [10], can affect processes such as migration, survival and reproduction [8], [11], [12]. An annual perspective allows a better approach to understanding how interactions among age, external forces, motion, and navigational capacities might be translated into patterns of movement [13]. Until recently, such data were almost impossible to collect; however, the development of appropriate individual tagging techniques has contributed to the appearance of such datasets.

Previous studies on fall migration in gulls has shown that the distance travelled throughout the individual's lifetime decreases with age [10], [14], [15]; individuals generally migrate shorter distances and winter closer to their breeding grounds as they get older. Moreover, it has been suggested that the onset of fall migration in young birds is characterized by shorter, exploratory flights [15]. However, as for many other studies of movement dynamics, the data came from recoveries of dead birds (e.g., from metal-ring numbers [14], [15]), or alternatively, movements were only reported for part of the annual cycle [16]–[18], thus narrowing the conclusions to a specific context. More importantly, migration movements were treated separately from other movements within the individual's life history [19].

However, geographic distribution patterns may not be exclusively a feature of the wintering period, and segregation among individuals within a population may occur year around, as hypothesized by Baker [14]. We therefore addressed these issues in the present study using a dataset based on re-sightings of individually color-ringed gulls (data from living individuals) to analyze, for the first time, the annual movements of Lesser Black-backed Gulls (*Larus fuscus*) throughout their lifetimes.

The analysis of birds' movement patterns [20], [21] is of fundamental importance, not only to increase our knowledge of their population dynamics, but also to identify important patterns of movements with potential implications for conservation plans [22], [23], population control [24] and/or the evaluation of human health risks (e.g., dissemination of contagious diseases [25]). *L. fuscus* is a widespread migratory species (i.e., with different populations facing different migratory/ecological problems) with a long-lifespan and a close association with man, and therefore represents an outstanding model to address all these issues.

The present study focused on the interactions between the individual's age and its pattern of movement throughout the annual cycle, by combining information on the individual's movement with information on its age (used as a measure of sexual maturation) at the time of each specific movement, with the aim of shedding light on the movement patterns of the gulls. Differences in movement patterns (short vs. long movements and/or distinct directionality) in relation to maturation stage (immature vs. matures) were analyzed.

## Materials and Methods

A total of 10,557 Lesser Black-backed Gulls were color-ringed as pulli at the Ribble Estuary (53°42′N, 02°55′W), Tarnbrook Fell (54°01′N, 02°35′W) and South Walney colonies (54°03′N, 3°12′W), in northwestern England between 1997 and 2007. The project run with permission of the British Trust for Ornithology on behalf of the UK authorities (permit number 4240), that conforms to the legal requirements for conservation and welfare. During this period, observations were collected throughout the population range to produce a dataset containing 19,096 observations (initial ringing date and place; re-sighting date and place).

Observations were grouped into age classes reflecting the different stages of sexual maturation and navigational experience: immature (0- and 1-year olds), 1st breeding year (2-year olds) and mature (older than 2 years). Gulls in the first breeding year demonstrate behavior patterns intermediate between those of immature and mature gulls [10], justifying their inclusion as a separate age class. The annual range variation among these classes of individuals was studied using a subset of observations that included only one randomly chosen recovery per individual. In addition, to avoid hypothetical biases towards one of the age classes, observations carried out at distances less than 15 km from the breeding colonies were excluded from the analysis. A total of 2,492 observations were used. Differences among the ranges of distribution of each age class throughout the annual cycle (from month-to-month) were analyzed using a two-factor ANOVA parametric test. Because, the variable ‘distance’ was not uniform, a logarithmic transformation was applied. A pot-hoc HSD test was used.

Differences in length and orientation of the movements among the different age classes were assessed by analyzing pairs of observations of the same individual within a 60-day period. The 60-day period result from direct analysis of Figure S1 where distance travelled was plotted in function of the number of days between two consecutive observations of the same individual (Fig S1). Note that there is no direct relationship between the number of days between two consecutive observations and distance travelled, and that although there are no differences in using a 30-day period or a 60-day period, the 60-day period allowed the analysis of a larger sample size (n = 342).

Distances between pairs of observations were calculated from the geographic coordinates using the orthodromic distance, which represents the distance between two points along a spherical surface. The orthodromic distance (*l*) was calculated using the expression (a), where lat1  =  the latitudinal coordinate of the location 1, lon1  =  the longitudinal coordinate of the location 1, lat2  =  the latitudinal coordinate of the location 2, lon2 =  the longitudinal coordinate of the location 2, and r =  the radius of the earth, 6,371 km [26].
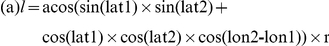



Movements were then divided into short movements (≤100 km; movements with a distance of 0 km were excluded) or long movements (>100 km). We also determined the bearings between the start and end locations for pairs of observations of the same individual using the expression (b):
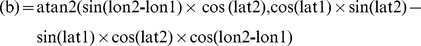



Bearings calculated using (b) were then grouped by age (immatures or adults), type of movement (short- or long-movement) and by month at the start of each movement. Data were analyzed using Oriana 2 software. The mean vector (α, mean angular direction; r, mean vector length) per month and per type of movement (short or long movement) was determined for each age group. Each mean vector (i.e., significance of the directional orientation of the individual movements) was tested for directional significance using the Rayleigh test [27].

## Results

**Figure 1:**
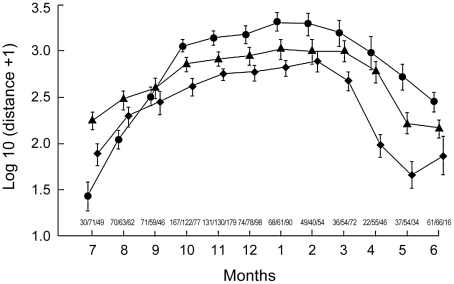
Annual distribution range of *Larus fuscus* by age classes. Mean values for distribution ranges are given by symbols. Diamonds, Class 0- and 1-year-old gulls (sexually immature); Triangles, 2-year-old gulls (first breeding year); Circles, 3 or more-years-old gulls (sexually mature). Bars, 95% confidence interval. At the bottom, sample sizes are given for each mean value in each month in the following order; class 0-1/class 2/class 3 or older.

Analysis of 19,096 re-sightings of over 10,000 individually ringed gulls (data collected between 1997 and 2007) revealed high seasonal variation throughout the annual cycle (Fig. 1). The results point to a clear effect of sexual maturation on the geographic distribution of gulls, with individuals in different sexual maturation classes exhibiting different annual ranges (Fig. 1). As gulls matured, the annual range distribution decreased and the mean of the age-class annual range shifted towards the north (i.e., closer to the breeding grounds). Results showed that the distance of all individuals to the breeding grounds changed significantly along the year (month effect, ANOVA: F_11,2456_ = 148.31, p<00001). Furthermore, the factor age class had a significant effect on the distance to the breeding grounds (age effect, ANOVA: F_2,2456_ = 136.29, p<00001) and both month and age class contributed to the distance of each individual to the breeding grounds (month×age, ANOVA: F_22,2456_ = 15.147, p<00001; see Fig 1). As a consequence of that interaction, the distances to the breeding area differed among immatures (0- and 1-year old), gulls in the 1st breeding year (2-years old) and mature gulls (3 or more years old) in all months (post hoc HSD test p<0,05), except for September.

**Figure 2:**
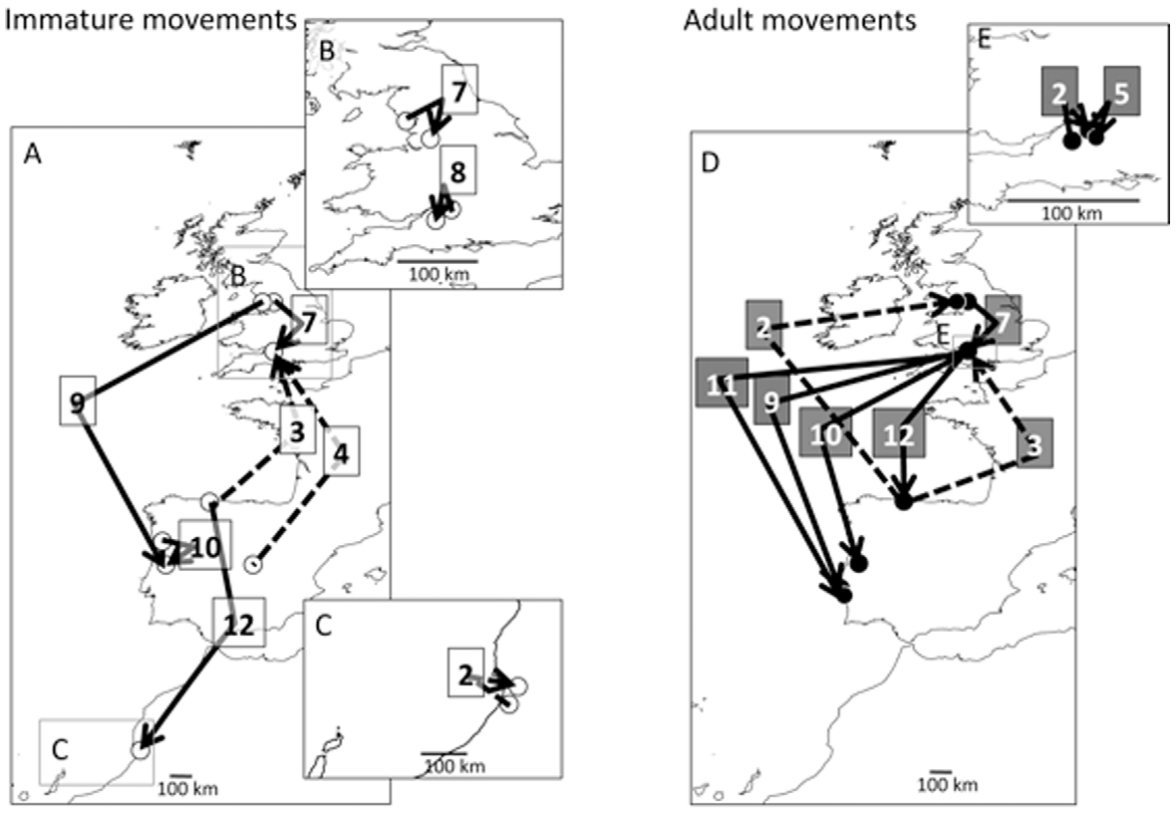
Examples of the most common movements observed across the population range. Median departure and arrival locations (circles) are connected by arrows indicating the direction of the movement. Numbers represent the month when the movement started (1 for January, up to 12 for December). A- Immatures long movements. B and C- Immatures short movements. D- Adults long movements. E- Adults short movements. Full line arrows, southward movements; dashed line arrows, northward movements.

Because distinct movement abilities can also influence movement patterns, movements were classified into two categories to assess how birds moved throughout the year, both during and between displacement phases. Interestingly, there was a large difference between the two spatial scales of movements (short movements and long movements; Table 1). Large scale movements (>100 km) were mostly associated with directional seasonal population movements (i.e., significant vector lengths; Table 1) linked to the annual cycle dynamics, between the wintering and breeding grounds, while short movement (≤100 km) were associated with non-directional movements (i.e., non significant vector length; Table 1). There were a few interesting exceptions to this general trend. Immature gulls presented significantly short directional movement towards the southeast in July (Table 1 and Fig. 2) prior to more southwards movements in August and September (Table 1 and Fig 2). Moreover directional movements in immatures also occurred in February. In adults, short directional movements only occurred in February and May (Table 1 and Fig. 2).

**Table 1:** Mean bearings of individual movements throughout the annual cycle.

LONG MOVEMENTS													
Month		JUL	AUG	SEP	OCT	NOV	DEC	JAN	FEB	MAR	APR	MAY	JUN
**0**–**1 years old**	n	78	4	6	31	11	4	1	3	4	5	4	9
	α	170.5°	160.9°	199.4°	184.9°	156.0°	205.8°	185.2°	182.4°	13.2°	13.1°	356.9°	244.0°
	r	0.97	0.55	0.97	0.75	0.37	0.86	1	0.83	0.94	0.96	0.48	0.23
	p	**0.0001**	ns	**0.002**	**0.0001**	ns	**0.04**	ns	ns	**0.017**	**0.003**	ns	ns
**3 or more years old**	n	3	2	6	6	18	7	4	8	6	1	0	0
	α	167.2°	203.1°	206.1°	205.0°	190.4°	213.0°	261.4°	13.0°	359.5°	346.0°	—	—
	r	0.99	1	0.86	0.99	0.7	0.90	0.1	0.62	0.98	1	—	—
	p	**0.034**	ns	**0.006**	**0.0001**	**0.0001**	**0.0001**	ns	**0.025**	**0.0001**	ns	—	—

SHORT MOVEMENTS													
Month		JUL	AUG	SEP	OCT	NOV	DEC	JAN	FEB	MAR	APR	MAY	JUN
**0**–**1 years old**	n	53	9	14	23	7	5	11	15	5	6	12	23
	α	130.2°	213.8°	91.2°	67.1°	122.9°	51.3°	142.2°	13.1°	245.9°	301.3°	104.7°	130.8°
	r	0.66	0.60	0.21	0.33	0.09	0.304	0.50	0.55	0.38	0.44	0.09	0.34
	p	**0.0001**	**0.037**	ns	Ns	ns	ns	ns	**0.008**	ns	Ns	ns	ns
**3 or more** **years old**	n	2	10	7	21	67	34	27	12	17	1	6	2
	α	231.1°	128.4°	217.0°	185.8°	349.0°	190.6°	61.4°	57.0°	12.9°	113.2°	198.3°	208.0°
	r	1	0.47	0.20	0.17	0.07	0.10	0.21	0.61	0.04	1	0.70	0.47
	p	ns	ns	ns	Ns	ns	ns	ns	**0.008**	ns	Ns	**0.048**	ns

Movement behavior of sexually immature (0- and 1-year-old) and mature (older than 3-year-old) Lesser Black-backed Gulls (*Larus fuscus*) at two spatial scales (short movements and long movements). n, sample size; α, mean vector angle; r, mean vector length. Text in bold indicates significance of the mean vector by the Rayleigh test.

## Discussion

Migratory species show seasonal variation in their distribution ranges; northern hemisphere species generally breed in the northern part and winter in the southern part of their distribution ranges [5], [6], [19]. The results of this study demonstrated that this annual range was greatly reduced in sexually mature individuals, with mature breeders shortening their migratory movements and remaining further north than the rest of the population throughout the year. In contrast to previous assumptions, the results showed that gulls were geographically segregated by age throughout the annual cycle, rather than showing a geographic age-related cline only in the wintering areas [10]. This asymmetric distribution results from a reduction in the annual range distribution of sexually mature gulls, and from the non-overlap of the cores of the distribution ranges of immature and mature gulls. To our knowledge, no similar asymmetric distribution has been clearly described in other avian species, though it may not be uncommon, especially in species with differential migration patterns [7], [28], [29] and/or delayed sexual maturation [30]. The interplay between an individual's age and changes in the environmental conditions is a key factor influencing the decision to move [3], and therefore, in understanding the differential annual movement patterns. Moreover, life history tradeoffs appear to underlie the observed differential movement patterns, with the effect of sexual maturation pressures potentially extending well beyond the breeding period.

In addition to age-related effects on the annual distribution range, we also found that age affects the way individuals move during migration. Different strategies can be employed during migration, involving short or long movements. Short movements are likely to be associated with local movements [31], rather than migratory movements, as shown by the random directions observed in most months (Table 1). Nonetheless, the short movements of immature gulls in July, August, and February showed clear directional orientations in agreement with the population migratory direction (Fig. 2), suggesting that they initiated their migration by short leaps. Further analysis of these movements showed that first-winter birds initially displaced from their colonies towards the west coast of the UK in July, followed by short southerly movements along the west coast of the UK during August and September (see short movements, Table 1 and Fig. 2), before finally venturing into the open sea (see long movements, Table 1 and Fig. 2). This phenomenon was geographically localized during the fall migration, while the movements were spread throughout the wintering quarters in a global movement towards the north during the spring migration (Table 1 and Fig. 2).

Evidence from other species has suggested the existence of distinct patterns of movement in terms of timing of migration [32], in the direction of the migratory movement [33], [34] or in the itinerary [35]. Although the presence of movement differences between juveniles and adults has been hypothesized [14], [15], [33], no clear evidence has so far been presented, and the results of the current study are therefore significant in demonstrating these differences in migratory behavior for the first time, using data from living individuals. Although immatures migrate longer distances, they initiate their migratory journey by short movements, subsequently changing to longer movements to accomplish their migratory journey. In contrast, adults use long movements, even though they travel shorter distances than juveniles. This could be because first-winter birds require time to gain experience before venturing on their migratory journey. Several studies in Sweden have reported that migratory passerines facing the sea as they arrived at the Falsterbo Peninsula during their fall migration, sometimes reversed their migratory direction [36], [37], emphasizing the importance of these ecological barriers on the birds' movement dynamics. Apart from the initial migratory step of first-winter birds, short movements are usually associated with non-directional movements [38], [39], while long movements are generally linked to migratory behavior, and therefore correspond to directional movements within the annual cycle [2], [40].

Overall, the results of this study show that sexual maturation and navigational experience can influence the observed differences in movement patterns demonstrated in this *L. fuscus* population. Thus, age and behavioral factors, among others, clearly biased movement patterns and distribution of individuals throughout their lifetime. The effect of age on the distribution of animals makes it an important factor that must be taken into consideration when modeling population dynamics [41]. It has implications for the development of successful conservation and population management policies [42], and for the evaluation of potential human health risks [42], [43], including the dissemination of contagious diseases such as avian influenza.

## Supporting Information

Figure S1 Distance travelled between two consecutive observations of the same individual.
(DOCX)
