# Supplementary material for: Differential Annual Movement Patterns in a Migratory Species: Effects of Experience and Sexual Maturation
Source: PLoS One. 2011 Jul 20;6(7):e22433. doi: 10.1371/journal.pone.0022433 (PMC3140515; doi:10.1371/journal.pone.0022433)
Supplement: Figure S1 — Distance travelled between two consecutive observations of the same individual. (DOCX) [file pone.0022433.s001.docx]

SUPPORTING INFORMATION for

Differential annual movement patterns in a migratory species: effects of experience and sexual maturation

Paulo E. Jorge *^1^*^,^ *^2^* , David Sowter*^3^* and Paulo A. M. Marques *^1^*^,^ *^4^*

Summary:

This file contains figure S1


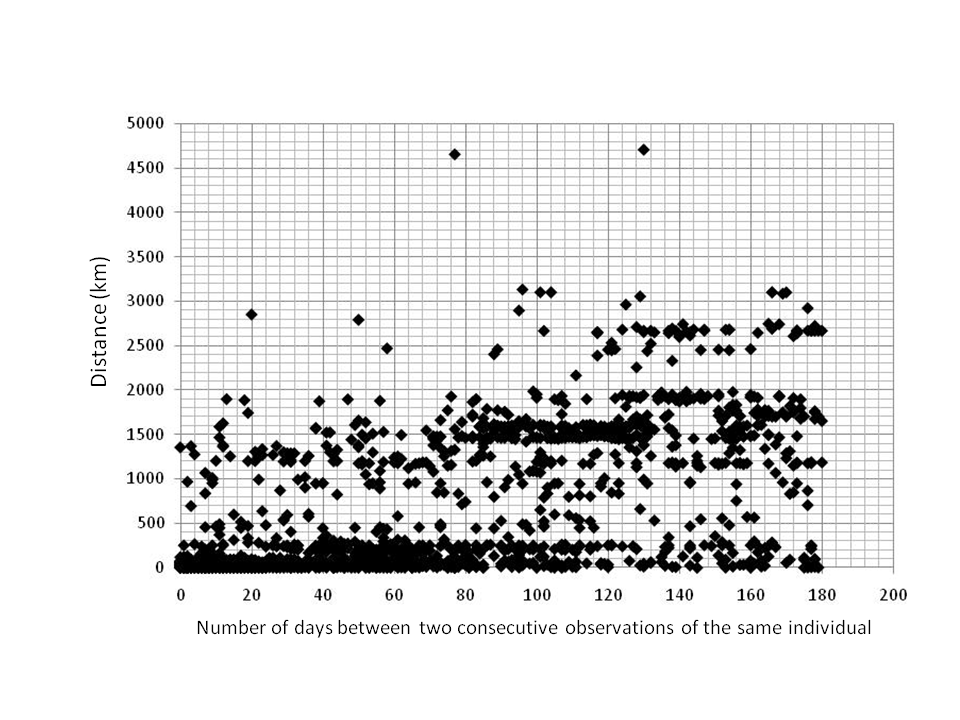


Figure S1

Distance travelled between two consecutive observations of the same individual.
